# The unique genetic variation within the O174L gene of Polish strains of African swine fever virus facilitates tracking virus origin

**DOI:** 10.1007/s00705-019-04224-x

**Published:** 2019-04-05

**Authors:** Natalia Mazur-Panasiuk, Grzegorz Woźniakowski

**Affiliations:** Department of Swine Diseases, National Veterinary Research Institute (NVRI), Partyzantów 57 Avenue, 24-100 Puławy, Poland

More than ten years after the emergence of African swine fever (ASF) in Georgia, the disease continues to pose a serious threat not only to European countries, which are leading pork producers, but also to worldwide trade of swine. The front of the ASF zone is constantly expanding westward, and recently, the first cases were reported in Belgium, suggesting a crucial role of human activity in ASF spread, considering the exceptional resistance of the causal agent to environmental conditions [1–4]. Moreover, the disease was confirmed in numerous pig herds in China, raising worldwide concern about an approaching ASF pandemic [5]. In Poland, the disease continues to spread into new areas. At the end of 2017, ASF cases were confirmed in wild boars in the area surrounding Warsaw, indicating an unexpected incursion of the disease into a previously ASF-free area of the country. From 2014 to the end of 2018, a total of 3347 cases in wild boars and 213 outbreaks in pig herds were reported in Poland [6].

Previous reports on the genetic diversity of African swine fever virus (ASFV) have reported a low level of genetic variability among European isolates [7–12]. Recent investigations of ASFV genotypes have been based on sequencing of the B646L and E183L genes and analysis of the intergenic region (IGR) between I73R and I329L genes [7, 8]. Another study of the molecular evolution of the EP402R and MGF505-2R genes of 67 Polish strains revealed minor genetic diversity within these genes, indicating slow but consistent molecular evolution in these regions [9].

Analysis of the whole genome sequences of seven Polish ASFV isolates revealed 14-nucleotide sequence variation within the O174L gene, which encodes the DNA polymerase beta-like protein [13]. Analysis of 46 ASFV isolates collected from ASF cases in wild boars and outbreaks in pigs between 2014 and 2018 in Poland confirmed the presence of a specific mutation – a 14-nucleotide insertion that might be useful to distinguish closely related ASFV isolates belonging to the highly pathogenic genotype II. This might aid in achieving a better understanding of ASFV transmission routes and sources of infection in Eastern and Central European countries. In this brief report, we present the identification of a new ASFV genetic marker within the nucleotide sequences of some ASFV Polish isolates. A spatiotemporal analysis in combination with phylogenetic data partially allowed the spread of ASFV in Poland to be traced.

Swine pulmonary alveolar macrophages (PAMs) were used to obtain and propagate 14 ASFV isolates for 3 passages. Total DNA was extracted from infected PAMs as described elsewhere [9], and viral DNA was amplified using an UPL real-time PCR method as described by Fernández-Pinero *et al.* [14]. Next, the whole genome was sequenced using a Miseq sequencer (Ilumina, San Diego, USA). The complete genome sequences of seven Polish ASFV isolates were submitted to the GenBank database under the accession numbers MG939583-MG939589. A genetic variation was observed within the O174L genes (encoding the beta protein of DNA polymerase) of three sequences collected in 2017 originating from two wild boars (cases #201 in Łosice and #754 in Piaseczno) as well as from domestic pigs (outbreak#81 in Radzyń Podlaski) from different locations. Subsequently, 46 ASFV isolates from the ASF National Reference Laboratory (NRL), collected between 2014 and 2018 (Table 1), were selected for further genomic analysis. In order to confirm the 14-nucleotide-insertion within the O174L gene, a pair of primers amplifying the 673-bp region of interest was designed. The primer binding sites were as follows: forward primer, 5’- TGGCTCAGACGATATTTCAACTC-3’, nt 128,160-128,182; reverse primer, 5’-GCCTCCACCACTTTGAACCAT-3’, nt 128,813-128,832. The optimal annealing temperature was 46°C. PCR was performed, and the products were subjected to sequencing by the Sanger method. The resulting sequences were aligned to those of closely related European genotype II isolates and those of other ASFV genotypes, with sequences available in the GenBank database. The following sequences were used for comparison (numbers in brackets indicate the: p72 genotype and the GenBank accession number): 47/Ss/2008 (I, KX354450.1), 26544/OG10 (I, KM102979.1), ASFV-SY18 (II, MH766894.1), ASFV/POL/2015/Podlaskie (II, MH681419.1), BA71V (I, NC_001659.2), Benin 97/1 (I, AM712239.1), E75 (I, FN557520.1), Estonia 2014 (II, LS478113.1), Georgia 2007/1 (II, FR682468.1), Georgia 2008/1 (II, MH910495.1), Georgia 2008/2 (II, MH910496.1), Kashino 04/13 (II, KJ747406.1), Ken05/Tk1 (X, KM111294.1), Kenya 1950 (X, AY261360.1), L60 (I, KM262844.1), Malawi Lil-20/1 (VIII, AY261361.1), Mkuzi 1979 (I, AY261362.1), NHV (I, KM262845.1), OURT88/3 (I, AM712240), Pretorisuskop/96/4 (XX, AY261363.1), R7 (IX, MH025917.1), Tengani 62 (V, AY261364.1), Warmbaths (III, AY261365.1), and Warthog (IV, AY261366.1). Additionally, two related reference DNA specimens from the NRL collection, Ukr12/Zapo and Lt14/1490, were also sequenced and included in the analysis. Nucleotide and amino acid sequence alignments were performed with a similarity cost matrix, using the Geneious R9 global alignment algorithm, and a phylogram was constructed using the minimum-evolution (ME) algorithm in MEGA 6 software (Fig. 1) [15]. All O174L gene sequences of Polish ASF viruses generated in this study were submitted to the GenBank database under the accession numbers MH764305-MH764350.

**Table 1:**
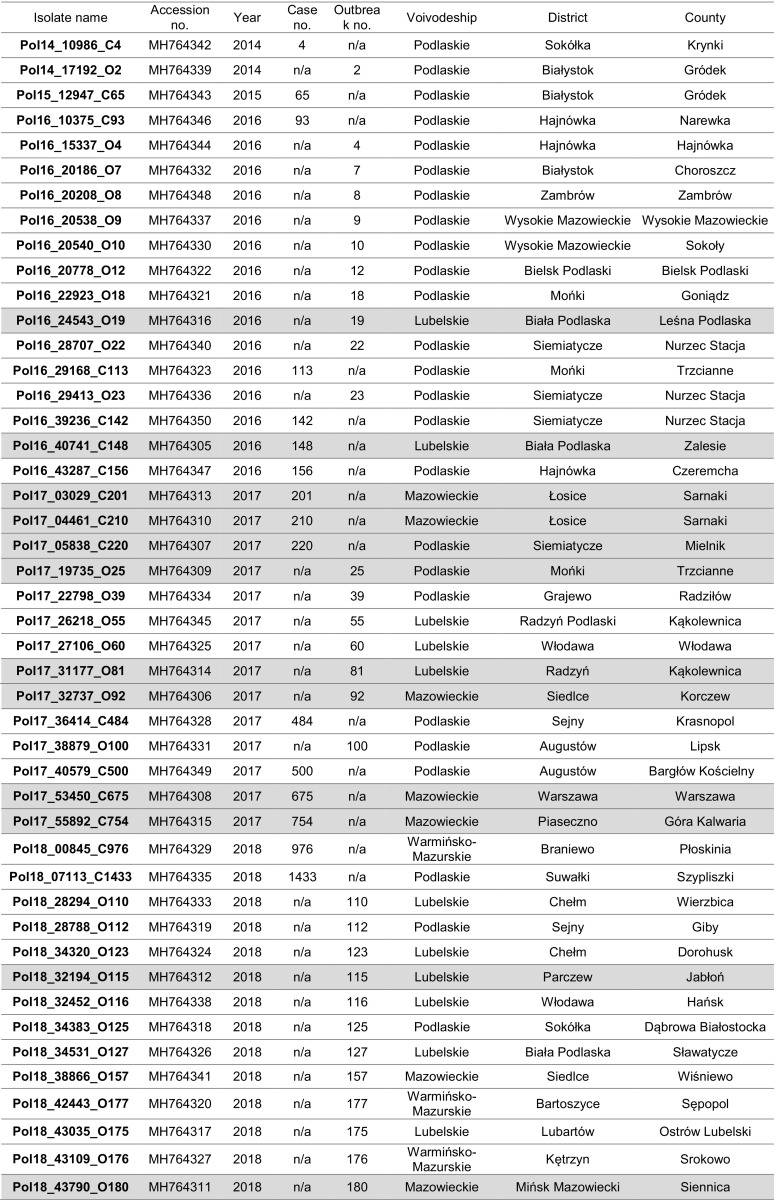
African swine fever isolates originating from Poland from 2014 to 2018, selected for a study of genetic variation within the O174L gene. Isolates containing a mutation resulting in the insertion of 14 nucleotides within the O174L gene are indicated by gray shading

**Fig. 1:**
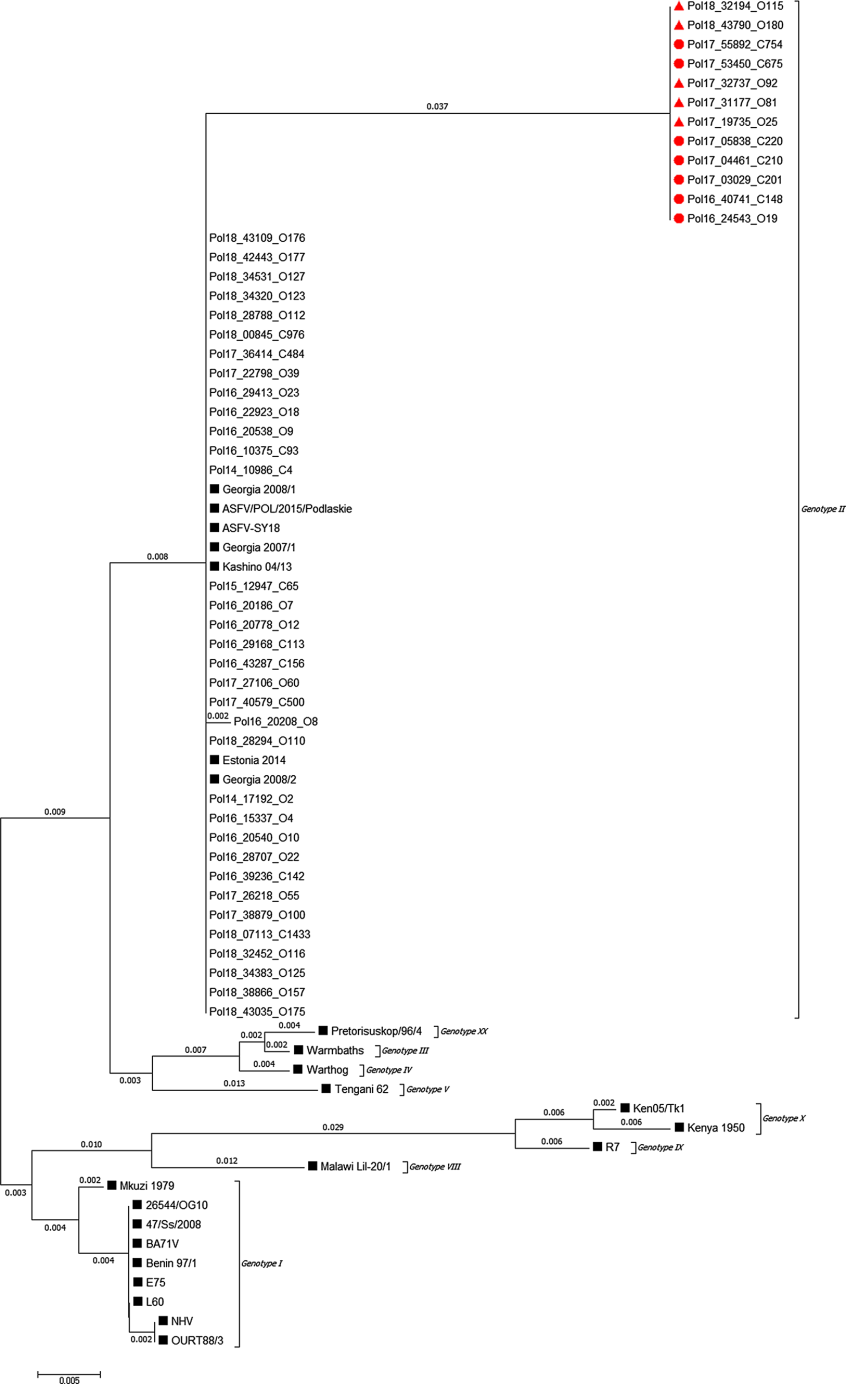
Minimum-evolution (ME) phylogenetic tree of African swine fever virus isolates from Eastern European strains based on the sequences of the O174L gene, including 46 sequences from Poland (Table 1) and 24 sequences of various genotypes. Black squares (■) indicate sequences available in the GenBank database and reference DNA from the NRL collection, which was also sequenced in this study. Red triangles and dots (▲, outbreaks, ●, cases) indicate samples containing the additional 14-nt insertion within the O174L gene. The scale bar indicates nucleotide substitutions per site.

An alignment of O174L nucleotide sequences of ASFV revealed the presence of an unique variation in 12 isolates originating from Poland. An insertion of 14 nucleotides (TCACTACTGAAAAA) was found, corresponding to a tandem repeat of nt 128282-128283 with reference to the Georgia 2007/1 strain. This insertion resulted in a shift to a different reading frame containing a premature stop codon. The wild-type and mutated proteins thus shared only 93% amino acid sequence identity. The disrupted O174L gene encodes a reparative DNA polymerase belonging to the family X DNA polymerases [16]. The ASFV polymerase X is able to efficiently repair single-nucleotide DNA breaks by base-excision repair (BER) during viral infection [17]. Previous studies have shown that deletion of the ASFV polymerase X gene results in accumulation of DNA damage with an increase of mutation frequency in swine macrophages [17]. Therefore, this particular gene is essential for maintaining viral genetic information. However, the effect of the observed insertion on the function of the polymerase remains unknown and needs to be investigated.

The 14-nucleotide-long insertion was found in 12 of the sequences listed in Table 1 and was found in isolates from wild boars (n = 6; designated as “C” [case] in the isolates names) as well as isolates from pigs (n = 6; designated as “O” [outbreak]). A spatiotemporal analysis using ArcGIS software showed that most of the ASFV isolates possessing the insertion were from the region near the Biała Podlaska district, where the mutation in O174L was first identified (outbreak #19, August 2016, 15 km from the Belarusian border) (Fig. 2). Seven other isolates collected between 2016 and 2018 originated from within approximately 50 km of outbreak #19. The remaining four isolates were from more distant locations: outbreak #25 (June 2016, Mońki district) 140 km from the Biała Podlaska district, and three isolates within the so-called “Warsaw cluster”, located between 100 and 130 km from outbreak #19. It is noteworthy that all three samples obtained from this particular cluster contained the mutation. Taking into consideration that wild boars have a rather sedentary life style and the fact that the emergence of ASFV within the “Warsaw cluster” was sudden, unexpected, and distant from the sites of previous disease reports, we presume that the virus was introduced into this particular area by humans [18]. Furthermore, the emergence of ASF in the Mońki district might also have resulted from human activity; however, only one out of four isolates tested (outbreak #18, case #113, outbreak #25 and outbreak #39) from this region contained the mutation, suggesting that ASFV was introduced into this particular district on at least two separate occasions. In 2018, 11 isolates originating from pig outbreaks were analyzed, of which only two contained the specific mutation. One of them was collected in the Parczew district, but five other isolates collected from 75 km away did not contain the mutation, again showing that multiple introductions had occurred in this region.

**Fig. 2:**
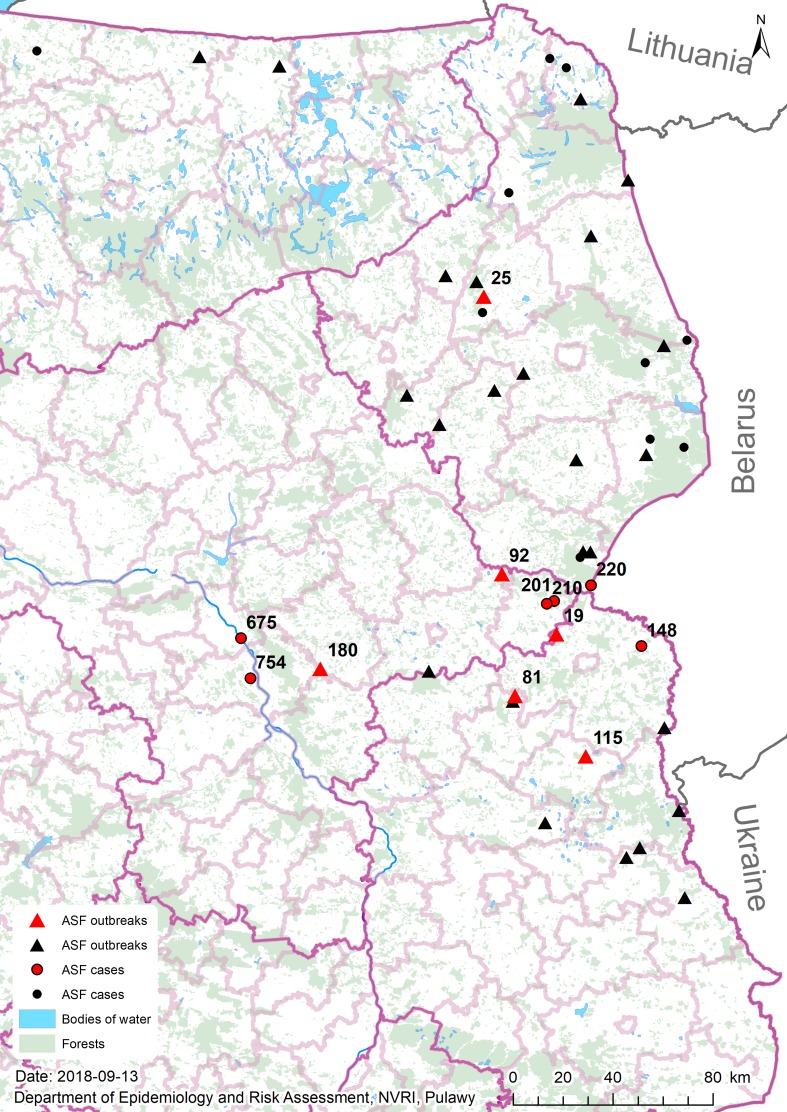
Geographical origin of the ASFV isolates investigated. Numbers correspond to the isolates containing the specific mutation, which are indicated in red (▲, outbreaks, ●, cases). Isolates without the mutation are shown in black and are not numbered.

Analysis of whole genome sequences of nine Polish ASFV isolates with confirmatory sequencing by the Sanger method revealed a new genetic marker within the O174L gene that might be useful for distinguishing closely related ASFV isolates in Poland. The data suggest that the simultaneous emergence of ASFV on domestic pig farms in various locations in Poland was probably due to multiple independent, unrelated disease outbreaks. However, an involvement of human activity in the spread of ASF in Poland, particularly into previously ASF-free and geographically distant areas could not be excluded, since the migratory activity of wild boars is very limited [18]. The genetic variability within the O174L gene provides an opportunity for deeper insight into the spread of ASFV in Poland and provides evidence of a relationship between geographically separated outbreaks, as was the case with the emergence of ASFV in the region surrounding Warsaw, where the virus seems to be to have been imported from the region of Biała Podlaska. The predicted amino acid protein sequence differs moderately from the original ASFV Georgia 2007/1 strain and shows only 93% sequence identity, whereas the encoded DNA polymerase is highly conserved among various ASFV genotypes (Fig. 1). The duplication of the 14-nt long sequence seems to have occurred suddenly, since no intermediate sequences between original and mutated sequences were detected, indicating that this tandem repeat has a higher evolution rate that the whole ASFV genome, a phenomenon that has been observed with other DNA viruses [19]. Genetic data like those presented in this report are essential for the study of ASFV evolution. However, an investigation of the effect of the variation described here on biology of the virus is still required.

## Data availability

The datasets generated and analyzed in the current study are available in the GenBank repository under accession numbers MH764305-MH764350.
